# The interplay of primer-template DNA phosphorylation status and single-stranded DNA binding proteins in directing clamp loaders to the appropriate polarity of DNA

**DOI:** 10.1093/nar/gku774

**Published:** 2014-08-26

**Authors:** Jaclyn N. Hayner, Lauren G. Douma, Linda B. Bloom

**Affiliations:** Biochemistry and Molecular Biology, University of Florida, Gainesville, FL 32610, USA

## Abstract

Sliding clamps are loaded onto DNA by clamp loaders to serve the critical role of coordinating various enzymes on DNA. Clamp loaders must quickly and efficiently load clamps at primer/template (p/t) junctions containing a duplex region with a free 3′OH (3′DNA), but it is unclear how clamp loaders target these sites. To measure the *Escherichia coli* and *Saccharomyces cerevisiae* clamp loader specificity toward 3′DNA, fluorescent β and PCNA clamps were used to measure clamp closing triggered by DNA substrates of differing polarity, testing the role of both the 5′phosphate (5′P) and the presence of single-stranded binding proteins (SSBs). SSBs inhibit clamp loading by both clamp loaders on the incorrect polarity of DNA (5′DNA). The 5′P groups contribute selectivity to differing degrees for the two clamp loaders, suggesting variations in the mechanism by which clamp loaders target 3′DNA. Interestingly, the χ subunit of the *E. coli* clamp loader is not required for SSB to inhibit clamp loading on phosphorylated 5′DNA, showing that χ·SSB interactions are dispensable. These studies highlight a common role for SSBs in directing clamp loaders to 3′DNA, as well as uncover nuances in the mechanisms by which SSBs perform this vital role.

## INTRODUCTION

Sliding clamps and clamp loaders play a crucial role in a variety of processes in DNA replication and repair. Because of their vital nature, sliding clamps and clamp loaders are present in all kingdoms of life (1). Sliding clamps are ring shaped proteins that are loaded around DNA in an adenosine triphosphate (ATP)-dependent reaction catalyzed by clamp loaders (1–3). Encircling DNA allows the sliding clamps to provide a mobile platform for DNA metabolic enzymes, most notably DNA polymerase, to bind and interact with DNA. Alone, the DNA polymerase has low processivity, and therefore frequently dissociates from DNA. In the presence of a sliding clamp, DNA polymerase is tethered to the parental DNA, preventing dissociation and increasing the rate of overall DNA synthesis (4,5).

The *Escherichia coli* and *Saccharomyces cerevisiae* sliding clamps have similar structural characteristics despite having little sequence similarity. Both clamps make a ring shape large enough to encircle DNA, and have similar features such as positively charged α-helical regions on the interior of the clamp that interact with DNA, and β-sheets on the outer surface (2,3). Both sliding clamps have an axis of symmetry through the center of the ring resulting in two distinct faces of the clamps, with a majority of protein·clamp interactions occurring on a single side via conserved hydrophobic pockets (6–8).

**Figure 1.**
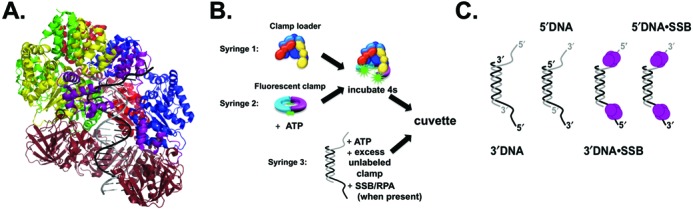
(**A**) DNA binding by clamp loaders. Duplex DNA spirals inside the cap of the clamp loader, with the single-stranded portion exiting between a gap in the clamp loader subunits (bacteriophage T4 clamp loader, PDB ID: 3U60 (15)). (**B**) A diagram showing the sequential mixing scheme in the stopped-flow. A solution of clamp loader in syringe 1, is mixed with fluorescent clamp and ATP from syringe 2 for 4 s to form an ATP·clamp loader·clamp complex. This complex is mixed with DNA, excess unlabeled clamp, and ATP. When SSB or RPA are present in reactions, they are included in syringe 3 with DNA to form SSB·DNA complexes. (**C**) Diagrams of the DNA structures used in these studies. All DNA structures are composed of two 60-mer oligonucleotides, annealed to produce a 30-nt duplex region, with two symmetrical 30-nt overhangs on either end of the duplex region. RPA was substituted for SSB in experiments with RFC and PCNA.

Because of the stable closed conformation of sliding clamps, ATPase enzymes called clamp loaders are required to load clamps around DNA. The *E. coli* clamp loader, γ complex, is a heptamer composed of three γ subunits, and one subunit each of δ and δ′ to form a core cap-like structure with χ and ψ as accessory subunits (9,10). The *S. cerevisiae* clamp loader, replication factor C (RFC), is a pentamer composed of Rfc1-5 subunits, which form a cap-like structure similar to the γ complex core (11). The cap-like clamp loaders interact with sliding clamps via the ‘bottom’ of the cap (see Figure 1A) (10–15).

Clamp loading reactions catalyzed by γ complex and RFC are similar in mechanism. Both enzymes are members of the AAA^+^ ATPase family, characterized by the use of ATP binding and hydrolysis to drive conformational changes that rearrange macromolecules (16). The γ complex binds and hydrolyzes three ATP molecules, one for each γ subunit (17–21). RFC can bind five molecules of ATP, but can only hydrolyze three to four, one in each of the Rfc2-4 subunits and potentially one in the Rfc1 subunit (22–24). ATP binding is required for both clamp loaders to bind the clamps and DNA with high affinity (22,25–28). DNA binding triggers ATP hydrolysis and results in release of the clamp·DNA complex (20,23,27,29).

Because sliding clamps are required by other proteins to stabilize interactions with DNA, clamp loaders must load clamps at the correct location on DNA for the downstream enzyme to work. Also, the clamp must be loaded in the correct orientation so that the face of the clamp needed for protein·protein interactions is accessible for binding. As an example, DNA polymerases bind the sliding clamp at primer/template (p/t) junctions with recessed 3′-ends for both DNA replication and repair. If the clamp is not at this location, or is facing the wrong direction, the polymerase will not bind in an effective manner required for DNA synthesis. At a replication fork, a new sliding clamp is needed every 2–3 s on the lagging strand for each Okazaki fragment. Because of this pressing timetable, the clamp loader must be able to quickly target the p/t junction and load a clamp.

It is still not clear which aspects of the p/t junction are recognized by the clamp loader to either promote clamp loading at the correct site, or inhibit clamp loading at the incorrect site. Recent crystal structures of both the *E. coli* and T4 bacteriophage clamp loaders in complex with p/t DNA give insight into DNA binding by the clamp loaders (Figure 1A) (14,15). In the crystal structure, the subunits of the clamp loader spiral in a pitch similar to DNA. The duplex portion of the p/t DNA sits inside the cap and presses up against the top of the cap, while the single-stranded overhang (SSO) exits the clamp loader through a gap between the δ and δ′ subunits in γ complex and the Rfc1 and Rfc5 subunits in RFC (14,15). As an example, Figure 1A shows a crystal structure of the T4 bacteriophage clamp loader in complex with the sliding clamp and DNA (PDB ID: 3U60) (15). In this structure, the duplex DNA can be seen inside the clamp loader cap, while the SSO exits in the gap between the A and A′ subunits (analogous to the gap between δ and δ′ in γ complex and the gap between Rfc1 and Rfc5 for RFC). Positively charged residues on the inside of the clamp loader cap make contacts with the phosphate backbone on the template strand in the duplex region of the p/t DNA. If this is the only contact made by the clamp loader to the DNA duplex, then it does not explain how the clamp loader can discern the correct from incorrect polarity of DNA. In fact, the opposite polarity of DNA was modeled into a crystal structure of γ complex, and the same contacts between the duplex DNA and the inside of the clamp were observed (30).

One possible method to target clamp loaders to the correct polarity of DNA is through interactions with single-stranded DNA binding proteins (SSBs) present at the p/t junction. These proteins may sense the orientation of DNA and confer this information to the clamp loaders. Another possible mechanism is that DNA specificity is mediated by sensing the charge of the p/t junction inserted in the clamp loader cap. If 5′DNA is present, a phosphate or triphosphate group will press up against the inside the cap, and this may be inhibitory to clamp loading. The clamp loaders must have a mechanism for discriminating between different DNA structures, because if clamp loading occurs on the incorrect polarity, the sliding clamp will not be available for interaction with downstream proteins.

Pre-steady state fluorescence-based clamp closing assays were used to examine how different DNA polarities affect clamp loading by γ complex and RFC. Two questions were asked for each clamp loader: first, are SSBs required to target clamp loaders to the correct polarity of DNA? The second question asked is: does the 5′phosphate (5′P) group at the p/t junction prevent clamp loading on the incorrect DNA polarity? These questions were addressed by measuring clamp loading on DNA of different polarities with and without SSBs. Additionally, DNA structures that lack a 5′P were also used. Results from both γ complex and RFC are compared to determine if there is a similar mechanism between the two clamp loaders, or if this step marks a divergence in function.

## MATERIALS AND METHODS

### Protein purification and storage

β was purified as described previously and stored in a buffer containing 20 mM Tris pH 7.5, 10% glycerol, 0.5 mM Ethylenediaminetetraacetic acid (EDTA) and 2 mM dithiothreitol (DTT) (19,31). For β closing assays, a β mutant was designed such that the native cysteine residues at position 260 and 333 were mutated to serine to prevent off-target labeling, and residues I305 and R103 at either side of the clamp interface were mutated to cysteine and labeled with AF488 (β-AF488_2_) (32). The individual subunits of γ complex (γ, δ, δ′, χ, ψ) were purified and reconstituted as described previously (19,33–36). The γ complex was stored in the same buffer as for β, but with the addition of 50 mM NaCl. The χ-less γ complex (γ_3_δδ′ψ) was provided by the M. O'Donnell laboratory.

Proliferating cell nuclear antigen (PCNA) was purified as described previously (37,38). PCNA was stored in 30 mM HEPES pH 7.5, 150 mM NaCl, 10% glycerol, 0.5 mM EDTA and 2 mM DTT. For the PCNA closing assay, the native PCNA cysteine residues at positions 22, 62, 81 were mutated to serine, while residues I111 and I181 at the interface were mutated to cysteine. These cysteine resides were labeled with AF488 (PCNA-AF488_2_) (39). To measure PCNA release, S43 was mutated to cysteine in the C22/62/81S background and labeled with MDCC (PCNA-MDCC) ((39) and Marzahn, submitted for publication).

RFC with a full-length Rfc1 subunit was expressed from a single plasmid (provided by the M. Hingorani laboratory) in *E. coli* BL21 (DE3) Rosetta cells (Millipore) for 8 h at 25°C (40). Cells were lysed by French press in 30 mM HEPES pH 7.5, 300 mM NaCl, 10% glycerol, 0.5 mM EDTA and 2 mM DTT. Purification steps included a HighTrap SP column, a HighTrap heparin column and finally a MonoQ column (all GE Healthcare). RFC was stored in the same buffer as PCNA, with the addition of 300 mM NaCl and 750 mM maltose for stability (41).

SSB was purified as described previously (42), and stored in the same buffer as γ complex. Purified replication protein A (RPA) was provided by the M. O'Donnell laboratory and was stored in the same buffer as PCNA.

### DNA structures

DNA oligomers (Integrated DNA Technology) were purified using 10% denaturing polyacrylamide gels. Oligomers were designed such that annealing resulted in p/t DNA structures with a 30-nucleotide duplex region, and symmetrical 30-nucleotide overhangs of either the 3′ or 5′ polarity (Figure 1C). Oligomer sequences for 3′DNA were: ATTATTTACA TTGGCAGATT CACCAGTCAC **ACGACCAGTAATAAAAGGGACATTCTGGCC** and CTTTCAGGTC AGAAGGGTTC TATCTCTGTT **GGCCAGAATGTCCCTTTTATTACTGGTCGT**. Oligomer sequences used for 5′DNA were: **CTTTCAGGTCAGAAGGGTTCTATCTCTGTT** GGCCAGAATG TCCCTTTTAT TACTGGTCGT and **AACAGAGATAGAACCCTTCTGACCTGAAAG** CGTAAGAATA CGTGGCACAG ACAATAGTCA. The DNA sequence regions shown in bold represent the complementary regions that form the duplex, while the sequence regions in normal type represent the single-stranded overhangs. These symmetrical DNA overhangs were designed to simplify kinetics by presenting the clamp loader with only ss/ds junctions, one on each side, as opposed to a structure in which one end is blunted while the other end is a ss/ds junction. Because the clamp·clamp loader has a footprint of ∼20 bp on p/t DNA (15,43,44), it is unlikely that multiple clamp·clamp loader complexes can bind to both ends of the DNA structure simultaneously.

### Pre-steady state stopped-flow

Pre-steady state clamp closing reactions were measured using an Applied Photophysics SX20 MV stopped-flow. Reactions were performed at 20°C with a 3.72-nm band pass. A sequential mixing scheme was used in which the clamp, clamp loader and ATP were mixed and incubated for 4 s before mixing with a solution of ATP, DNA and excess unlabeled clamp to initiate the reaction (Figure 1B). In reactions containing SSB or RPA, these SSBs were included in the syringe with DNA so that the clamp loader·clamp complex was added to a SSB·DNA complex. Final concentrations of reaction components were 20 nM clamp loader, 20 nM fluorescent clamp, 40 nM DNA, 0.5 mM ATP, 200 nM unlabeled clamp and 400 nM SSB or RPA unless otherwise noted. AF488 was excited at 490 nm and emission was measured using a 515 nm cut-on filter. MDCC was excited at 425 nm and emission was measured with a 455 nm cut-on filter. Reactions with γ complex and β had final buffer conditions of 20 mM Tris pH 7.5, 50 mM NaCl, 8 mM MgCl_2_, 4% glycerol, 0.5 mM EDTA and 2 mM DTT. Reactions with RFC and PCNA had final buffer concentrations of: 30 mM HEPES pH 7.5, 150 mM NaCl, 10 mM MgCl_2_, 10% glycerol, 100 mM maltose, 0.5 mM EDTA and 2 mM DTT.

To calculate the observed rates of clamp loading, pre-steady state time courses for β-AF488_2_ closing and PCNA-MDCC release were fit to a single exponential decay (Eq. 1), while pre-steady state PCNA-AF488_2_ closing time courses were fit to a double exponential decay (Eq. 2). Pre-steady state PCNA-AF488_2_ closing time courses with 5′DNA·RPA were fit to an exponential increase and decrease (Eq. 3), and rates and amplitudes were 3.0 ± 1.2 s^−1^ and 0.48 for the increase and 0.09 ± 0.01 s^−1^ and 1.9 for the decrease. Values reported represent the average of three independent experiments with standard deviation, unless otherwise noted.

(1) \begin{equation*} y = a(e^{ - k_{{\rm obs}} t} ) + c \end{equation*}

(2) \begin{equation*} y = a_{{\rm fast}} (e^{ - k_{{\rm fast}} t} ) + a_{{\rm slow}} (e^{ - k_{slow} t} ) + c \end{equation*}

(3) \begin{equation*} y = a(1 - e^{k_{{\rm up}} t} ) + a(e^{ - k_{{\rm down}} t} ) + c \end{equation*}

## RESULTS

### SSB strongly aids in γ complex discrimination between 3′ and 5′-recessed ends of DNA

In order to be effective for DNA replication and repair, β must be loaded onto p/t junctions with a recessed 3′-end (3′DNA). Therefore, γ complex must have a mechanism to preferentially target this p/t junction instead of a p/t junction with a recessed 5′-end (5′DNA). Additionally, the single-stranded (ss) regions of these structures are coated with SSB *in vivo*. To determine how both the structure of DNA and the presence of SSB contribute to DNA specificity by γ complex, a fluorescent β closing assay was used to report on productive clamp loading events (45). Briefly, clamp closing is measured using a β mutant labeled with AlexaFluor 488 (AF488) on either side of the interface. When β-AF488_2_ is closed, the fluorophores at the interface are in close enough proximity to self-quench. When γ complex opens β-AF488_2_, the fluorophores move apart relieving the quenching, thus leading to an increase in AF488 intensity. X-ray crystallography showed that the Cys mutations and fluorophores do not affect the β-clamp structure, and activity assays showed the mutations/fluorophores do not affect interactions with γ complex (32). Figure 1B shows the sequential mixing stopped-flow scheme used for this assay. The content of syringe 1, γ complex, is mixed with the contents of syringe 2, β-AF488_2_ and ATP. This solution is incubated for 4 s to allow a γ complex·ATP·β-AF488_2_ complex to form. The clamp loading reaction is then initiated by mixing this solution with the contents of syringe 3: DNA, ATP, excess unlabeled β and SSB (when present). The decrease in fluorescence of AF488 as the clamp closes was monitored as a function of time. Final concentrations of the components are 20 nM γ complex, 20 nM β-AF488_2_, 0.5 mM ATP, 40 nM DNA, 200 nM unlabeled β and 400 nM SSB (when present). The unlabeled clamp serves as a chase to limit the fluorescent closing reaction to a single turnover. The four different DNA structures used throughout this study are shown in Figure 1C. Observed rates of β closing were calculated from the time courses using an exponential decay equation (Eq. 1). In cases where clamps are productively loaded, time courses are not simple exponentials due to kinetic steps including DNA binding and ATP hydrolysis which occur prior to the closing step that is monitored. However, the exponential fit provides a reasonable estimate of the rate of change of fluorescence.

**Figure 2.**
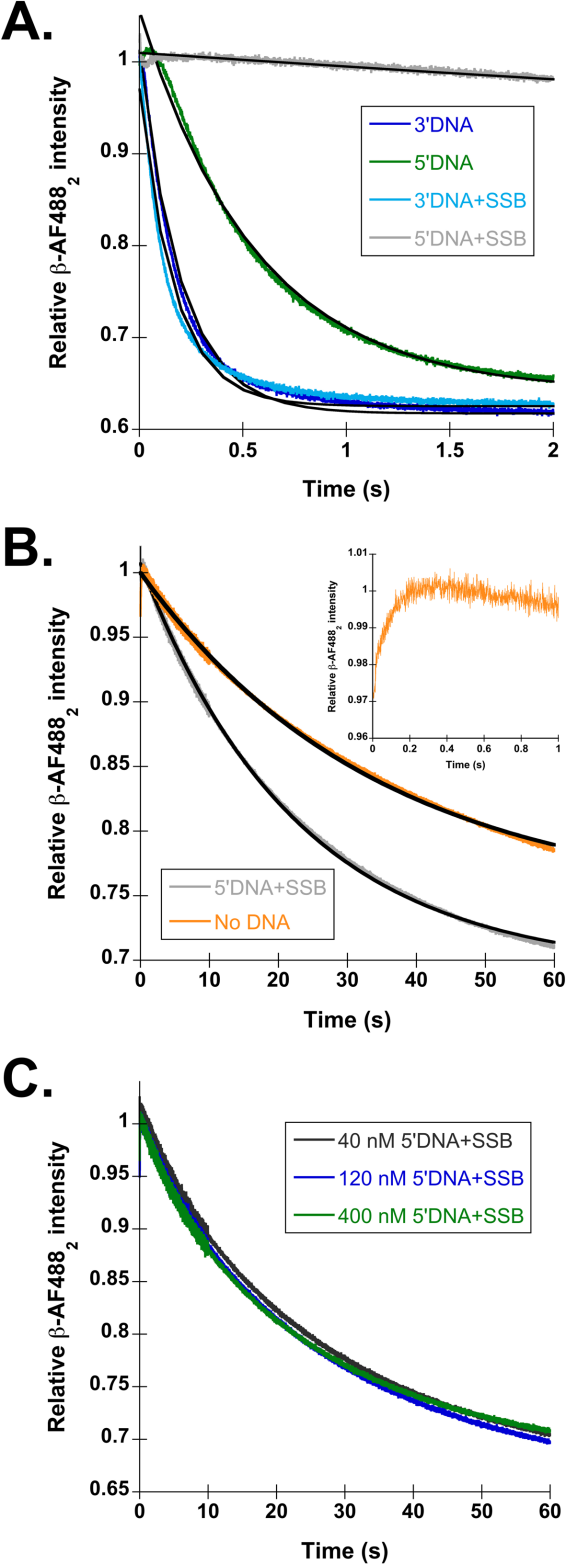
SSB inhibits β closing on the incorrect polarity of DNA. (**A**) Representative time courses for β-AF488_2_ closing on each DNA substrate shown in Figure 1C. The traces are color coded as follows: 3′DNA (dark blue), 5′DNA (green), 3′DNA·SSB (light blue) and 5′DNA·SSB (gray). The time courses were fit (black lines through the traces) to single exponential decays (Eq. 1), and observed rates calculated from these fits are reported in Table 1. (**B**) β closing in reactions with 5′DNA·SSB (gray) and in the absence of DNA (orange). Inset shows the time course for clamp loading in the absence of DNA on a 1 s timescale to highlight the increase in signal observed at the beginning of the time course. (**C**) β closing is shown as a function of 5′DNA·SSB concentration. The DNA and SSB concentrations used were: 40 nM DNA and 320 nM SSB (black), 120 nM DNA and 960 nM SSB (dark blue) and 400 nM DNA and 3.2 μM SSB (green).

Representative β closing traces with 3′DNA (dark blue) and 5′DNA (green) are shown in Figure 2A, with calculated observed rates reported in Table 1. Closing on 3′DNA was about twice as fast as 5′DNA, 4.6 ± 0.4 s^−1^ compared to 1.9 ± 0.3 s^−1^. This small difference in rates of clamp closing indicates that there is not strong discrimination between the two polarities of DNA at this step in the clamp loading reaction. Because clamp closing is a downstream step in the clamp loading cycle, it is possible that 5′DNA has a larger effect on the rate of an upstream step, such as DNA binding or ATP hydrolysis. Nevertheless, 5′DNA still triggers an overall clamp loading reaction that is only 2-fold slower than the reaction triggered by 3′DNA.

**Table 1.** Calculated observed rates of β-AF488_2_ closing with wild type γ complex

DNA structure	−SSB	+SSB
	*k*_obs_ (s^−1^)^a^	*k*_obs_ (s^−1^)^a^
3′DNA	4.6 ± 0.4 (2.4)	5.7 ± 0.3 (95)
3′DNA, no 5′P	6.2 ± 1.3 (3.3)	8.7 ± 0.6 (145)
5′DNA	1.9 ± 0.3 (1)	0.06 ± 0.01 (1)
5′DNA, no 5′P	2.5 ± 0.3 (1.3)	0.18 ± 0.04 (3)
No DNA	0.03 ± 0.01	N/A

^a^Rates relative to the rate of 5′DNA are given in parentheses to show the fold change.

N/A indicates no reaction performed. Observed rates are calculated from Equation (1) and represent the average and standard deviation of three independent experiments.

One reason why there is not a large difference observed between 3′ and 5′ DNA may be that naked DNA is not sufficient to confer polarity information to the γ complex; the γ complex may receive additional polarity cues from SSB. To test this hypothesis, the same fluorescent assay was used to measure β closing on 3′ and 5′DNA, but this time with the addition of 400 nM SSB to the reactions. Figure 2A shows representative β closing time courses for 3′DNA·SSB (light blue) and 5′DNA·SSB (gray), with calculated observed rates reported in Table 1. The presence of SSB has very little effect on the rate of clamp closing on 3′DNA, with observed rates of 4.6 ± 0.4 s^−1^ in the absence of SSB, and 5.7 ± 0.3 s^−1^ in the presence of SSB. However, SSB greatly reduces the observed clamp closing rate on 5′DNA by 30-fold compared to naked 5′DNA, and by 100-fold compared to 3′DNA·SSB. This strong, 100-fold difference, between 3′ and 5′DNA supports the hypothesis that SSB confers DNA specificity to γ complex, by inhibiting clamp loading on 5′DNA.

Clamp closing with 5′DNA·SSB is so slow, that it behaves as if ATPγS is present (45) or no DNA is present in the reaction at all. In the presence of ATPγS or in the absence of DNA, the γ complex·β simply dissociates in a passive manner. Based on the similarity of time courses and observed rates for β closing on 5′DNA·SSB and β closing in the absence of DNA (Figure 2B and Table 1), reactions with 5′DNA·SSB likely occur predominantly via passive dissociation rather than an active clamp loading mechanism. DNA binding by γ complex·β is a bimolecular reaction, so reactions with 5′DNA·SSB may appear as if no DNA is present because clamp loader affinity for this DNA substrate is very weak and only a small fraction of clamp loaders productively bind DNA. If this is the case, then increasing the concentration of 5′DNA·SSB should increase DNA binding by the clamp loader, and increase the fraction of clamps that close in an active clamp loading reaction. To test this possibility, the concentration of 5′DNA·SSB was increased by as much as a factor of 10 in the β closing assay, but there was no increase in the closing rate at higher 5′DNA·SSB concentrations (Figure 2C). These data suggest that either γ complex·β is unable to bind 5′DNA·SSB, or it binds 5′DNA·SSB in a conformation that is incompatible with promoting active clamp loading.

On a technical note, the first 50–100 ms of each β closing time course contain a small increase in signal (Figure 2B, inset), but the source of the change is unclear (32,45). This small increase in fluorescence is also present in reactions that lack DNA, suggesting that it does not stem from interactions with DNA or conformational changes upon DNA binding. Control stopped-flow experiments using a single mixing scheme were performed to determine whether the small increase in signal is due to rapid mixing of the proteins in the stopped-flow apparatus, or due to a dilution of γ complex·β as the two syringes are mixed. Supplementary Figure S1A shows a time course in which pre-formed γ complex·β in syringe 1 was mixed with an equal concentration of pre-formed γ complex·β in syringe 2. In this case, the increase in signal in the first second of the reaction was absent, showing that high-velocity movement of the pre-formed γ complex·β through the stopped-flow is not the cause of the signal change. Supplementary Figure S1B shows a time course in which pre-formed γ complex·β in syringe 1 was mixed with buffer in syringe 2. This 2-fold dilution in the clamp loader·clamp complex produced the small increase in fluorescence. The signal change is unlikely to be due to dissociation of the clamp loader·clamp complex on dilution because this would produce a closed clamp with reduced fluorescence. One possible reason for this dilution effect is that the rapid dilution changes the β clamp dimer equilibrium.

### RPA contributes to RFC discrimination between the two polarities of DNA

The requirement for sliding clamps to be loaded at 3′-recessed ends is not unique to *E. coli*. In eukaryotes, the RFC clamp loader must load PCNA at 3′DNA for various DNA replication and repair functions. To determine the contribution of DNA structure and SSB (RPA) to RFC specificity, the closing assay described above for β was adapted for PCNA to measure productive PCNA loading by RFC. PCNA was selectively labeled with AF488 on either side of the interface so that when PCNA-AF488_2_ is open, the fluorophores are highly fluorescent, but when PCNA-AF488_2_ is closed, the fluorophores self-quench, leading to a decrease in signal. Control experiments with the PCNA-AF488_2_ clamp indicate that the fluorophores do not affect interactions with RFC (39). The relative decrease in AF488 fluorescence intensity is smaller for PCNA-AF488_2_ than β-AF488_2_ because the fluorophores are not completely quenched when the clamp is closed and the structures of the clamps and corresponding positions of the fluorophores are not identical. The background level of fluorescence is greater for PCNA with three labeled interfaces than β with two, which contributes to the smaller change in relative intensity. The PCNA-AF488_2_ assay was used to measure clamp closing triggered by different polarities of DNA (Figure 1C) to see how RFC discriminates between DNA structures. The stopped-flow mixing scheme shown in Figure 1B was used with RPA added in syringe 3 when present.

**Figure 3.**
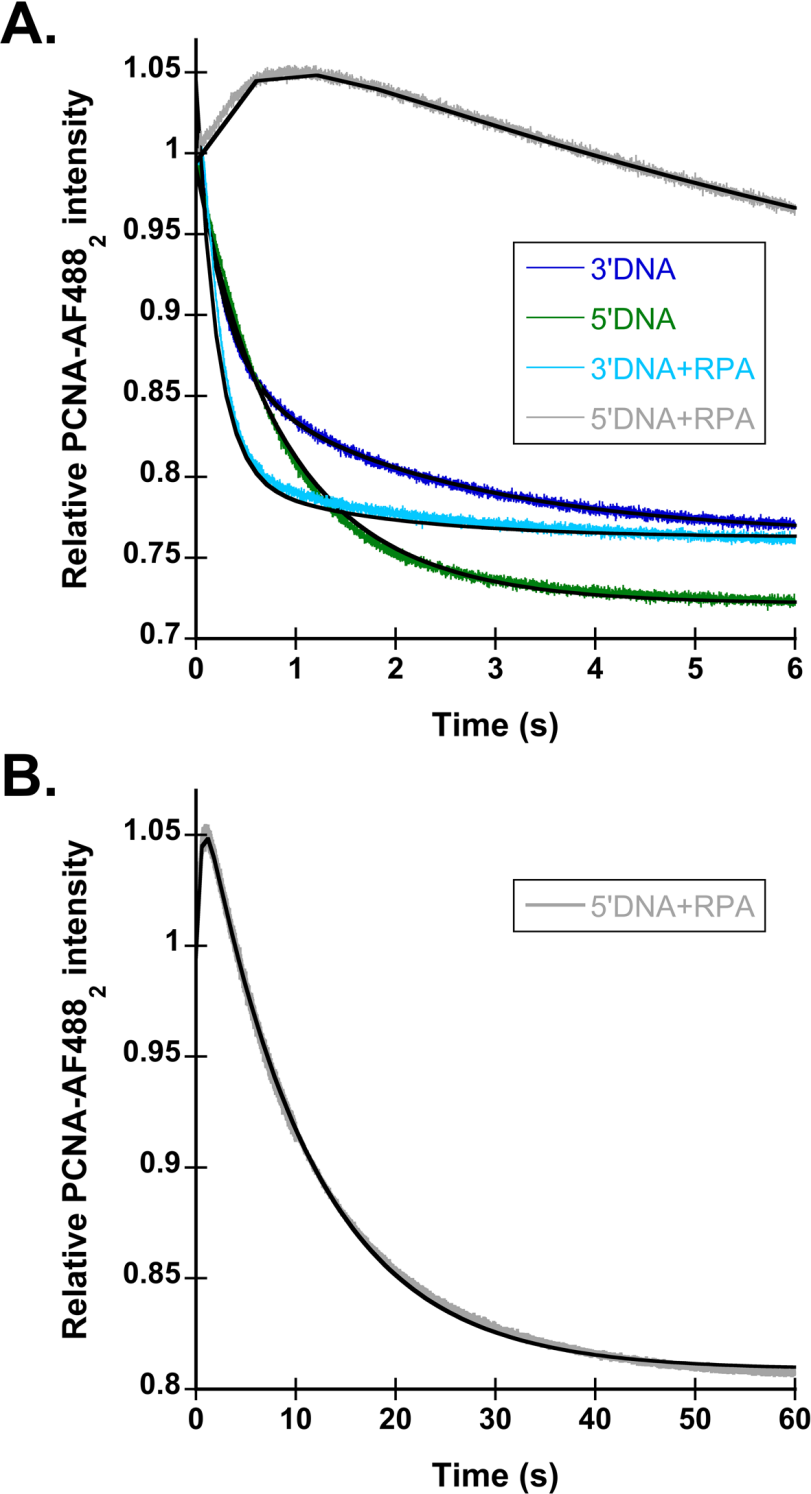
RPA inhibits PCNA loading on the incorrect polarity of DNA. (**A**) Representative time courses for PCNA-AF488_2_ closing are shown using the DNA structures shown in Figure 1C. The 3′DNA is shown in dark blue, 5′DNA is shown in green, 3′DNA·RPA is shown in light blue and 5′DNA·RPA is shown in gray. PCNA-AF488_2_ closing was performed as described in Figure 1B, with final concentrations of components: 20 nM RFC, 20 nM PCNA-AF488_2_, 40 nM DNA, 0.5 mM ATP and 200 nM unlabeled PCNA. The time courses were fit to double exponential decays (Eq. 2), except for 5′DNA·RPA, which was fit to an exponential increase and decrease (Eq. 3). The black lines through the time courses represent the results of this fit, and observed rates calculated from this fit are reported in Table 2. (**B**) PCNA-AF488_2_ closing with 5′DNA·RPA, on a longer time scale.

The decreases in PCNA closing time courses were biphasic and were fit to a double exponential decay (Eq. 2) to estimate observed rates (Figure 3A). One explanation for these biphasic kinetics is that there are two populations of clamp loader·clamp complexes that close PCNA at different rates upon binding DNA. This possibility is unlikely as the rates and amplitudes of the two phases remain consistent between different preparations of RFC. Another explanation for the biphasic kinetics is that the fluorescence of AF488 is sensitive to two different interactions, interactions between the two adjacent fluorophores that self-quench on clamp closing, and interactions between RFC and the fluorophore that enhance fluorescence in the bound state and decrease on release. In this case, the faster phase (*k*_obs1_) would represent the decrease in fluorescence as the PCNA-AF488_2_ clamp closes, while the second, slower phase (*k*_obs2_), would represent the decrease in fluorescence as the PCNA-AF488_2_ clamp is released from RFC. This second hypothesis is supported by directly measuring rates of PCNA release using an independent PCNA binding assay. Briefly, PCNA was labeled with MDCC on the surface to which RFC binds, and the fluorescence of the RFC·PCNA-MDCC complex is greater than the fluorescence of unbound PCNA-MDCC ((39), and M. Marzahn, submitted for publication). The observed rates of PCNA release, measured via the PCNA-MDCC assay, mirror the slow rates (*k*_obs2_) measured in closing reactions for each of the DNA substrates with and without bound SSB (Supplementary Figure S2 and Table S1).

Figure 3A shows representative PCNA-AF488_2_ closing time courses using 3′ (dark blue) and 5′DNA (green) structures with observed rates reported in Table 2. Only the faster PCNA closing rate (*k*_obs1_) is referenced in the text, because this phase of the reaction that likely represents PCNA-AF488_2_ closing, however, both rates are reported in Table 2. As seen with γ complex, PCNA closing on 3′DNA was only 2–3-fold faster than closing on 5′DNA, with observed rates of 3.3 ± 0.15 s^−1^ compared to 1.3 ± 0.08 s^−1^, respectively. This small difference in rates indicates that, like γ complex, RFC cannot markedly discriminate between the two polarities of naked DNA at this step in the clamp loading reaction.

**Table 2.** Calculated observed rates of PCNA-AF488_2_ closing with RFC

DNA substrate	−RPA		+RPA	
	*k*_obs1_ (s^−1^)^a^	*k*_obs2_ (s^−1^)	*k*_obs1_ (s^−1^)^a^	*k*_obs2_ (s^−1^)
3′DNA	3.3 ± 0.2 (2.5)	0.44 ± 0.01	4.4 ± 0.5 (49)	0.33 ± 0.07
3′DNA, no 5′P	2.9 ± 0.3 (2.2)	0.44 ± 0.07	4.6 ± 0.7 (51)	0.26 ± 0.02
5′DNA	1.3 ± 0.1 (1)	0.27 ± 0.03	0.09 ± 0.01 (1)	N/A
5′DNA, no 5′P	1.2 ± 0.1 (0.9)	0.29 ± 0.03	0.79 ± 0.04 (8.8)	0.35 ± 0.02

^a^Rates relative to the rate of 5′DNA are given in parentheses to show the fold change.

N/A indicates no observed rate. Observed rates are calculated from Equations (2) and (3) (5′DNA·RPA only) and represent the average and standard deviation of three independent experiments.

To determine whether RPA enhances specificity of RFC for 3′DNA, PCNA-AF488_2_ closing assays were performed in the presence of 400 nM RPA. The addition of RPA to 3′DNA did not change the observed rates of time courses significantly (Table 2 and Figure 3A); the observed rate for PCNA closing in the absence of RPA was 3.3 ± 0.2 s^−1^, while the observed rate in the presence of RPA was 4.4 ± 0.5 s^−1^. Therefore, RPA does not affect PCNA closing on the correct, 3′DNA structure. Addition of RPA to 5′DNA had two effects on reaction time courses: the overall rate of PCNA closing was decreased dramatically with a monophasic rather than biphasic decrease in fluorescence, and a small increase in fluorescence occurred at early times. This trace was fit to an exponential increase and decrease (Eq. 3) to obtain the observed rate of the small increase in the beginning as well as the observed rate of the decrease as PCNA-AF488_2_ closes. Only the exponential decay rate is reported in Table 2 and referenced in the text. It is unclear what causes the increase in signal at the beginning of this time course, as it is not present in the time courses with other DNA substrates. The PCNA-AF488_2_ closing rate on 5′DNA·RPA is the same as the PCNA-MDCC release rate (Supplementary Figure S2. and Table S1.) indicating that PCNA is closing as it is released by RFC. Overall, RPA decreased the PCNA closing rate on 5′DNA by 20-fold compared to naked 5′DNA, and 50-fold compared to 3′DNA·RPA. A longer time scale for closing with 5′DNA·RPA is shown in Figure 3B. Based on these data, RPA confers DNA polarity specificity to RFC. The targeting of clamp loaders to 3′DNA by SSBs appears to be a conserved mechanism between RFC and γ complex.

### RPA and a 5′ phosphate on the primer strand are required for robust inhibition of PCNA loading on 5′DNA, but SSB is the main requirement for inhibition of β loading on 5′DNA

The p/t junction sits inside the cap of the clamp loader (see Figure 1A), pushing up against the top of the clamp loader (14,15). The presence of a 5′ phosphate (5′P) group on 5′DNA, either alone or in conjunction with the SSBs, may inhibit clamp loading on the incorrect polarity of DNA. To test this hypothesis, the DNA substrates in Figure 1C were synthesized without 5′P groups, and clamp closing experiments were performed as described above (Figure 1B).

**Figure 4.**
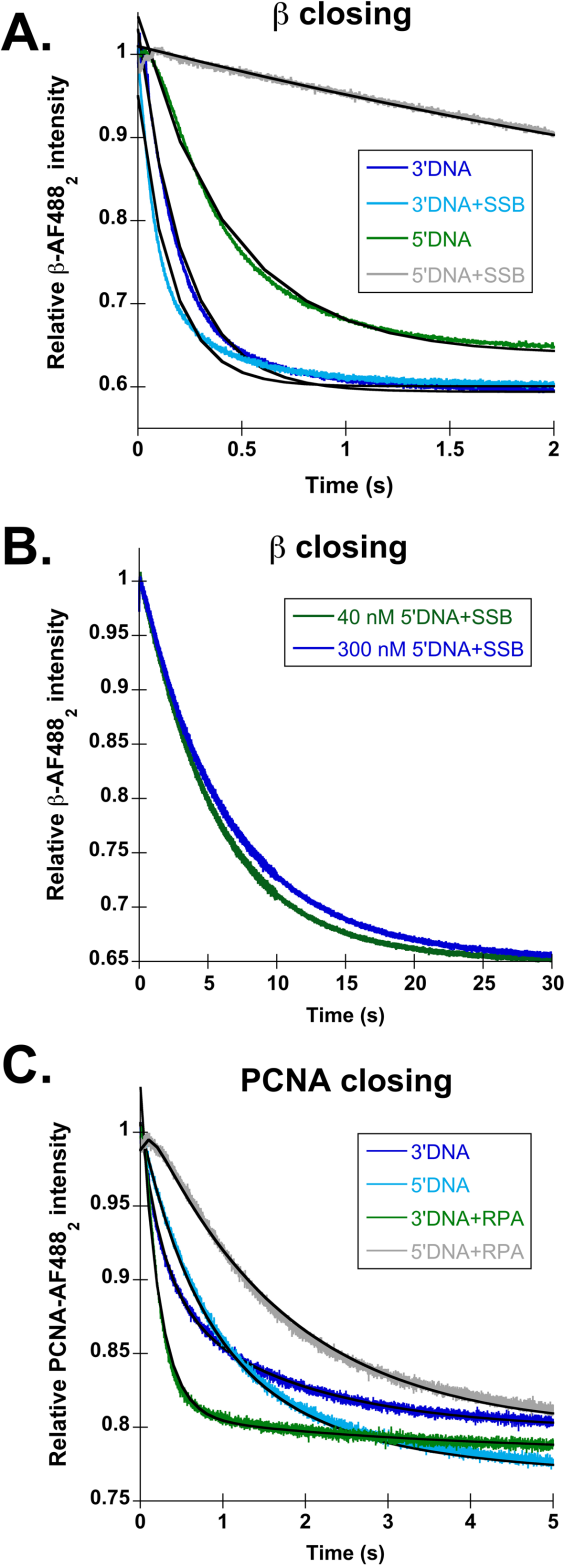
A 5′ phosphate (5′P) group is required for RPA-dependent inhibition of PCNA-AF488_2_ closing on 5′DNA, but to not for SSB-dependent inhibition of β-AF488_2_ closing on 5′DNA. Reactions were performed as outlined in Figure 1B. DNA structures are illustrated in Figure 1C, with the exception of the 5′P group, which is not present here. (**A**) Representative β-AF488_2_ closing traces are shown for 3′DNA (dark blue), 5′DNA (green), 3′DNA·SSB (light blue) and 5′DNA·SSB (gray). Calculated observed rates of β-AF488_2_ closing are reported in Table 1. (B) β closing is shown as a function of 5′DNA·SSB concentration. The DNA and SSB concentrations used were: 40 nM DNA and 320 nM SSB (green), 300 nM DNA and 3 μM SSB (dark blue). (C) Representative PCNA-AF488_2_ closing time courses are shown for 3′DNA (dark blue), 5′DNA (green), 3′DNA·RPA (light blue) and 5′DNA·RPA (gray). Calculated observed rates of PCNA-AF488_2_ closing are reported in Table 2.

β-AF488_2_ closing time courses for 3′ (dark blue) and 5′DNA (green) without 5′P groups are shown in Figure 4A, with observed rates reported in Table 1. Removing the 5′ phosphorylation did not have a large effect on the rates of clamp loading on ‘naked’ DNA; β-AF488_2_ closing remained about 2-fold faster on 3′ than 5′DNA. SSB was added to these DNA structures to determine if 5′ phosphorylation status contributes to inhibition of β loading on 5′DNA by SSB. Figure 4A shows time courses for clamp loading on 3′DNA·SSB (light blue) and 5′DNA·SSB (gray) without the 5′P groups. The observed rate for β closing on 3′DNA·SSB increased slightly over the 5′ phosphorylated 3′DNA·SSB. The observed rate for 5′DNA·SSB was 3-fold faster than 5′ phosphorylated 5′DNA·SSB and about 6-fold faster than passive dissociation with no DNA (Table 1). To determine whether the increase in the closing rate on unphosphorylated 5′DNA·SSB was due to an increase in DNA binding, the closing reaction was repeated at a higher 5′DNA·SSB concentration (Figure 4B). There was no difference in rates at the two 5′DNA·SSB concentrations showing that the rate of clamp closing is not dependent on the rate of DNA binding under these conditions. This suggests that removing the 5′P from 5′DNA·SSB affects the rate of an intramolecular reaction in the clamp loader·clamp·DNA complex. Although the closing rate increased for unphosphorylated 5′DNA·SSB, there is still a 50-fold difference in observed closing rates between 3′DNA·SSB and 5′DNA·SSB showing that SSB is the most important factor in inhibiting β loading onto 5′DNA, and the 5′P group plays a lesser role.

The contribution of 5′ phosphate groups to DNA substrate specificity was also addressed for PCNA loading by RFC. Figure 4C shows PCNA-AF488_2_ closing time courses for 3′DNA (dark blue) and 5′DNA (green) with observed rates reported in Table 2. There is no difference in closing rates on phosphorylated and unphosphorylated DNA in the absence of RPA; closing rates remain 2–3-fold faster on 3′DNA than 5′DNA. Addition of RPA to these DNA structures yielded a surprising result. In the absence of a 5′P group, the observed rate of PCNA closing for 3′DNA·RPA (Figure 4C, light blue) was the same as if the 5′P group was present. However, for unphosphorylated 5′DNA·RPA structures (Figure 4C, gray), RPA no longer strongly inhibited PCNA loading. When RPA is present, there was a 50-fold difference in closing rates for phosphorylated 3′ and 5′DNA, but only a 6-fold difference for the unphosphorylated DNA substrates. This shows that the ability of RPA to inhibit PCNA loading on 5′DNA is dependent on the presence of a 5′P group at the p/t junction. This is in stark contrast to results for γ complex and β, where SSB strongly inhibited β-AF488_2_ loading on 5′DNA whether or not the 5-end was phosphorylated.

### The χ subunit of γ complex is required to inhibit clamp loading on unphosphorylated 5′DNA·SSB, but is dispensable for inhibition on phosphorylated 5′DNA·SSB

Based on the data above, SSBs enhance the specificity of clamp loaders for 3′DNA over 5′DNA. Given that the χ subunit of the γ complex has been implicated in mediating interactions with SSB (46,47), the contribution of the χ subunit to γ complex specificity was investigated. Closing reactions were performed with the two DNAs differing in polarity in the presence and absence of SSB (Figure 1C) using χ-less γ complex (γ_3_δδ′ψ); average observed rates calculated from three independent experiments are shown in Table 3.

**Table 3.** Calculated observed rates of β-AF488_2_ closing with ‘χ-less’ γ complex (γ_3_δδ′ψ)

DNA structure	−SSB	+SSB
	*k*_obs_ (s^−1^)^a^	*k*_obs1_ (s^−1^)^a^
3′DNA	5.2 ± 0.2 (3.0)	1.8 ± 0.3 (30)
3′DNA, no 5′P	6.1 ± 0.4 (3.6)	2.7 ± 0.1 (45)
5′DNA	1.7 ± 0.1 (1)	0.06 ± 0.02 (1)
5′DNA, no 5′P	1.8 ± 0.2 (1.1)	1.0 ± 0.2 (17)

^a^Rates relative to the rate of 5′DNA are given in parentheses to show the fold change.

Observed rates are calculated from Equation (1) and represent the average and standard deviation of three independent experiments.

**Figure 5.**
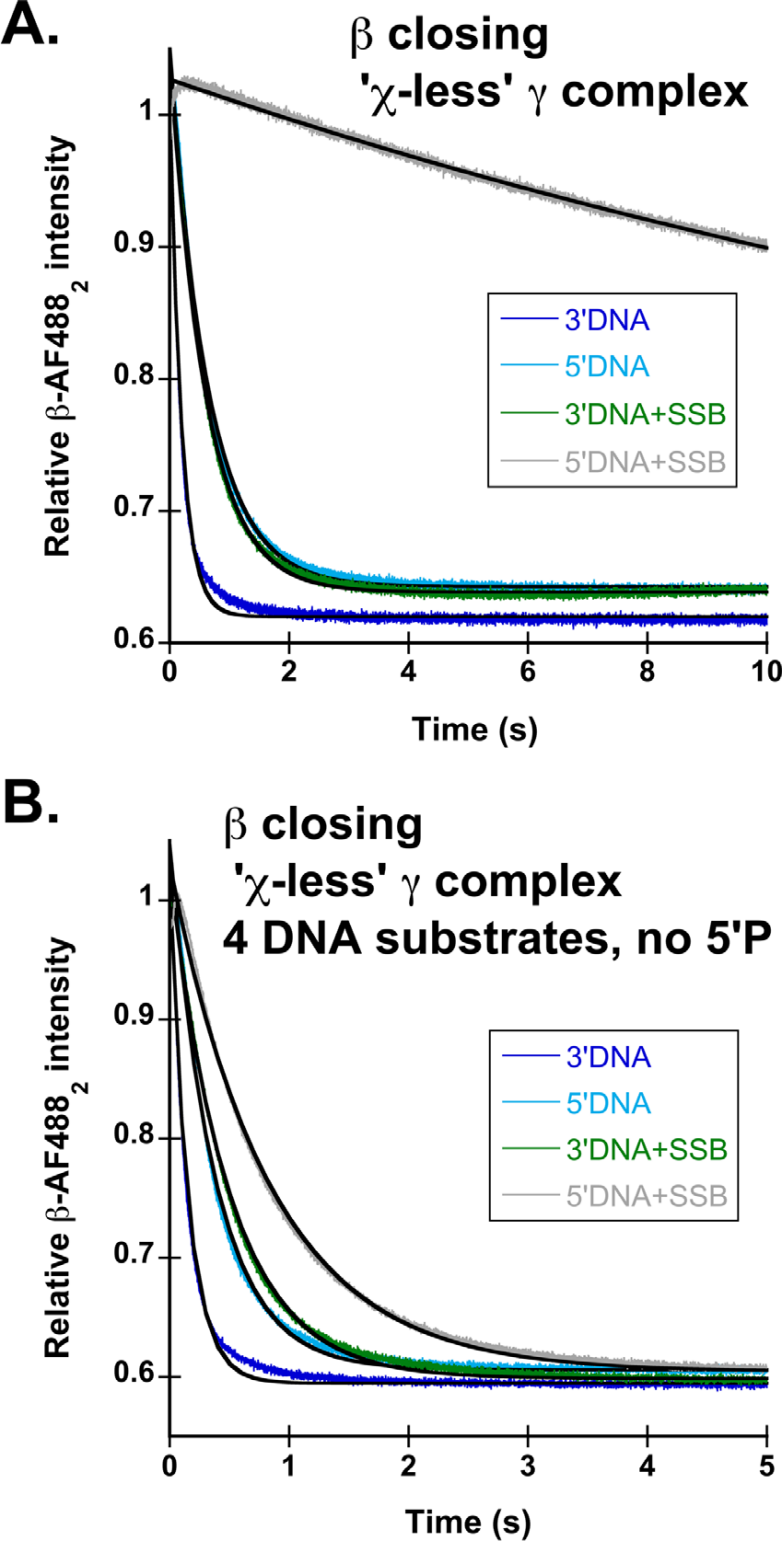
The χ subunit of γ complex is required for SSB-mediated inhibition of clamp loading on unphosphorylated 5′DNA, but is dispensable in the presence of a 5′P. Reactions were performed as outlined in Figure 1B. DNA structures used are those outlined in Figure 1C: 3′DNA (dark blue), 5′DNA (green), 3′DNA·SSB (light blue) and 5′DNA·SSB (black). Calculated observed rates for β-AF488_2_ closing using all DNA structures both with and without the 5′P are reported in Table 3. (**A**) Representative time courses for β-AF488_2_ closing with χ-less γ complex (γ_3_δδ′ψ) on phosphorylated DNA. (**B**) Representative time courses for β-AF488_2_ closing with χ-less γ complex on unphosphorylated DNA.

Observed β closing rates using χ-less γ complex in the absence of SSB are the same as rates for wild-type γ complex (γ_3_δδ′ψχ) whether or not the DNA is phosphorylated on 5'-ends (Figure 5A and B, dark blue and green), showing that the χ subunit is not required for the clamp loading reaction in the absence of SSB (48). Addition of SSB to 3′DNA both with and without the 5′P group slows β-AF488_2_ closing 2–3-fold by χ-less γ complex compared to wild type γ complex (Figure 5A and B, light blue). This demonstrates that the χ subunit enhances clamp loading on SSB-coated 3′DNA, but it is not absolutely required for this process. Interestingly, on 5′DNA·SSB, two different results are observed depending on whether the 5′-end is phosphorylated. In the presence of a 5′P group, the observed rate for clamp closing is the same for clamp loaders with and without the χ subunit (Figure 5A, gray), demonstrating that inhibition of clamp closing on 5′DNA by SSB is not dependent on the presence of a χ subunit. In contrast, removal of the 5′P group from 5′DNA·SSB increases the observed rate of β-AF488_2_ closing nearly 20-fold to 1.0 ± 0.2 s^−1^ for the χ-less clamp loader (Figure 5B, gray). This diminishes the difference between clamp closing rates on unphosphorylated 3′DNA·SSB and 5′DNA·SSB to about 3-fold, whereas there is a 30-fold difference in these rates for 5′-phosphorylated DNA. Taken together, the clamp closing experiments with wild-type and χ-less γ complex show that either a 5′P on DNA or the χ subunit on the clamp loader is required for robust inhibition of clamp closing on 5′DNA·SSB. When both the 5′P and χ are absent, inhibition of clamp closing is modest.

### The presence of non-cognate single-stranded binding proteins inhibit clamp loading on 5′DNA by γ complex, but to a lesser extent for RFC

**Figure 6.**
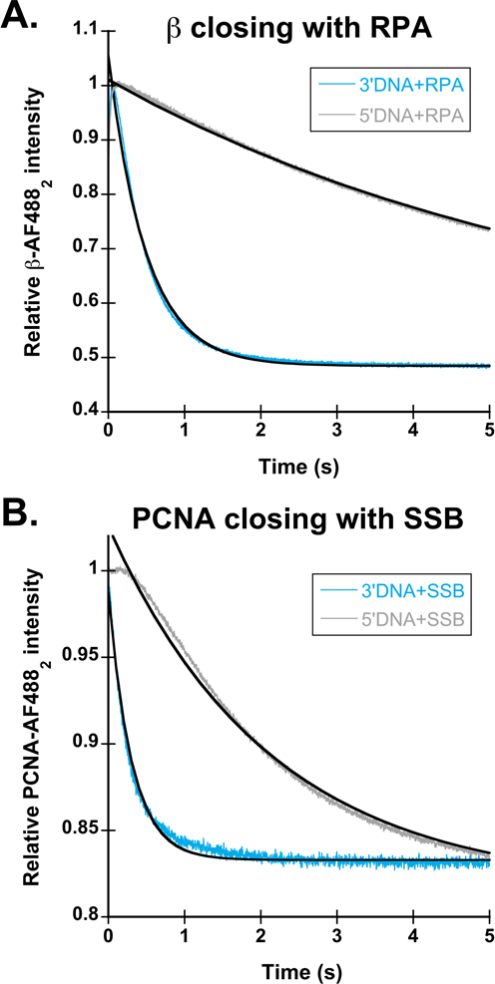
Clamp loading in the presence of non-cognate single-stranded binding proteins. (**A**) β closing reactions were performed with 3′DNA (light blue) and 5′DNA (gray) in the presence of RPA. Observed rates for clamp loading are the average of two experiments. Clamp loading on 3′DNA·RPA had an observed rate of 1.6 s^−1^, while 5′DNA·RPA had an observed rate of 0.12 s^−1^. (**B**) PCNA closing reactions were performed with 3′DNA (light blue) and 5′DNA (gray) in the presence of SSB. Observed rates for clamp loading are the average of two experiments. Clamp loading on 3′DNA·SSB resulted in a biphasic fluorescent decrease with *k*_obs1_ of 4.6 s^−1^ and a *k*_obs2_ of 1.8 s^−1^while 5′DNA·SSB time courses had only a single phase with an observed rate of 0.45 s^−1^.

The presence of SSB and RPA confers DNA specificity to γ complex and RFC, respectively. However, is it possible for RPA to confer specificity to γ complex or SSB to confer specificity to RFC? To test this, clamp closing reactions were performed for each clamp loader in the presence of the non-cognate SSBs. Figure 6A shows time courses in which β closing was measured with 3′DNA (light blue) and 5′DNA (gray) coated with RPA. Clamp closing by γ complex on 5′DNA·RPA (*k*_obs_ = 0.12 s^−1^) is about 13-fold slower than clamp closing on 3′DNA·RPA (*k*_obs_ = 1.6 s^−1^), showing that RPA enhances the specificity of γ complex for 3′-recessed ends over naked DNA. However, the magnitude of the difference is not as great as for SSB-bound DNA where the rate of clamp loading on 3′DNA·SSB (*k*_obs_ = 5.7 s^−1^) is nearly two orders of magnitude faster than the rate of clamp loading on 5′DNA·SSB (*k*_obs_ = 0.06 s^−1^). On the correct polarity DNA, clamp closing by γ complex is slower on 3′DNA·RPA than 3′DNA·SSB, and the magnitude of this difference, about a 3-fold decrease, is similar to the decrease in β closing rates on 3′DNA·SSB for the χ-less γ complex. This suggests that species-specific interactions between γ complex and SSB mediated by the χ subunit enhance clamp loading on DNA of the correct polarity, but are not required.

To determine if RPA specifically inhibits clamp loading by RFC on 5′DNA, or if SSB can substitute, PCNA closing reactions were performed with 3′DNA (light blue) and 5′DNA (gray) in the presence of SSB (Figure 6B). As with the *E. coli* clamp loader, there was about a 10-fold difference in rates of clamp closing on 3′ and 5′DNA with the non-cognate SSB. The rate of PCNA closing on 5′DNA·SSB was 0.45 s^−1^ compared to 4.6 s^−1^ on 3′DNA·SBB. In contrast to the *E. coli* clamp loader, non-cognate SSB binding to 3′DNA had no effect on the observed rates of clamp loading, with a *k*_obs1_ of 4.4 s^−1^ for 3′DNA·RPA and 4.6 s^−1^ for 3′DNA·SSB. This shows that unlike γ complex, species-specific interactions between RFC and single stranded binding proteins do not enhance the rate of clamp loading on 3′DNA; RFC can load clamps equally as well on 3′DNA in the presence of SSB or RPA. Although SSB decreased the PCNA closing rate on 5'DNA by an order of magnitude, it did not inhibit clamp loading as strongly as RPA did (Figure 3B), therefore, species-specific interactions between RFC·PCNA and RPA are required for robust inhibition of clamp loading on 5′DNA.

## DISCUSSION

Clamp loaders are required to load sliding clamps at nucleic acid ss/ds junctions to be used by DNA polymerases and other enzymes required for DNA replication and repair. The sliding clamps serve as a mobile platform to anchor or recruit these enzymes to DNA. Given that sliding clamps have two distinct faces, one of which is responsible for most protein·protein interactions, the orientation of the clamp at the ss/ds junction is important. Clamps are oriented appropriately when loaded at ss/ds junctions with 3′-recessed ends (3′DNA), thus clamp loaders must have a method for targeting 3′DNA over a junction with a 5′-recessed end (5′DNA). When sliding clamps are needed for DNA replication, there is an additional demand to find these 3′DNA junctions quickly, as a new sliding clamp is needed every 2–3 s for each Okazaki fragment. Clamp loaders must be able to bind and open the sliding clamp, find the correct DNA substrate, and load the clamp around DNA all within a few seconds. In this study, the question of how clamp loaders target the ss/ds junctions with 3′-recessed ends was addressed.

### Clamp loading on the correct polarity of DNA is unaffected by SSBs, but clamp loading on the incorrect polarity of DNA is inhibited by SSBs

Results presented in this work show that SSBs play an important role in clamp loader discrimination between the correct (3′DNA) and the incorrect (5′DNA) polarity by preferentially inhibiting clamp loading on 5′DNA. In the absence of the SSBs, neither γ complex nor RFC showed a strong preference for loading on 3′DNA over 5′DNA (Figures 2A and 3A, dark blue and green). But, in the presence of SSBs, clamp closing on 3′DNA was unaffected, while clamp closing on 5′DNA decreased 1.5–2 orders of magnitude for both γ complex and RFC (Figures 2A and 3A, light blue and gray). Previous work addressing the mechanism of DNA specificity by the γ complex used a method in which SSB was required to be present on the DNA substrates at all times, therefore the presence and absence of SSB could not be experimentally tested (30). To determine what factors contribute to this preferential inhibition of clamp loading on 5′DNA, effects of phosphorylation of 5′-ends and specific clamp loader–SSB interactions were examined.

### The role of phosphorylation of 5′-ends on clamp loading

Phosphorylation of the 5′-recessed end at ss/ds junctions plays a small role in SSB-mediated inhibition of clamp loading by γ complex. When a 5′P group is present, SSB inhibits clamp closing on 5′DNA such that closing likely occurs via a passive dissociation mechanism. When the 5′P was removed, the rate of clamp closing on 5′DNA increased by a factor of three (Figure 4A, gray), but was still nearly 50-fold slower than closing on 3′DNA (Figure 4A, light blue). Although the 3-fold increase in closing rate is not a large difference, it suggests that the mechanism of clamp closing may change from a passive release to an active but slow DNA-triggered reaction. Clamp closing rates did not increase with increasing concentrations of 5′DNA·SSB for either the phosphorylated or unphosphorylated DNA substrates (Figures 2C and 4B) suggesting that slow closing rates are not due to weaker DNA binding. Instead, the clamp loader likely interacts with 5′DNA·SSB differently than 3′DNA·SSB such that clamp closing is inhibited on 5′DNA·SSB. Interestingly, phosphorylation of the 5′-end plays a larger role in SSB-mediated inhibition by the χ-less γ complex. Removal of the 5′P group alleviated SSB-dependent inhibition of clamp loading by χ-less γ complex on 5′DNA such that the difference in clamp closing on 3′DNA and 5′DNA was about the same with and without SSB. As for the χ-less γ complex, the phosphorylation status of the 5′-end of the ss/ds junction is an important factor in the inhibition of PCNA loading on 5′DNA by RPA (Figure 4C). RPA resulted in about a 50-fold preference for 3′DNA when the 5′-end was phosphorylated, but only a 6-fold preference when the 5′-end was unphosphorylated. In the absence of RPA, the 5′ phosphorylation status had no effect on the closing rates suggesting that the 5′P itself is not responsible for inhibiting PCNA loading. Taken together, these results suggest that the 5′P influences the way that SSB and RPA bind to the single-stranded overhang to inhibit clamp loading by the χ-less γ complex and RFC.

### Contribution of species-specific clamp loader·single-stranded binding protein interactions to specificity to 3′-recessed ends

Both SSB and RPA bind ssDNA with a defined polarity via conserved oligonucleotide binding domains (49–51) and this would orient the SSBs differently on the ss regions of 3′DNA and 5′DNA. RPA is a heterotrimer such that binding to ssDNA overhangs would result in different subunits adjacent to the ss/ds DNA junction. The N-terminal domain of the Rpa1 subunit is oriented toward the 5′-end of ssDNA, while the Rpa3 subunit is oriented toward the 3′-end of ssDNA (50,51). Extrapolating this to ss/ds DNA junctions, a 3′DNA structure would have the Rpa3 subunit oriented closest to the ss/ds DNA junction, while 5′DNA would have the Rpa1 subunit oriented closest to the junction. This difference in orientation may give rise to differences in clamp loader–RPA interactions that target PCNA to 3′-recessed ends and target 9-1-1 clamp to 5′-recessed ends (52–54). Although the *E. coli* SSB is tetramer of identical subunits, the polarity with which ssDNA binds each monomer is such that different residues in the DNA binding domains are present at the 3′- and 5′-ends of ssDNA to create different interaction sites for proteins such as the clamp loader. However, a previous study showed when reverse polarity linkages were made to change the polarity of the ssDNA relative to the ss/ds DNA junctions, the *E. coli* clamp loader still showed a preference for 3′-recessed ends (30). In addition to the structured DNA binding domain, *E. coli* SSB contains a region of about 65 amino acids at the C-terminal end that is dynamic and/or unstructured (55,56). The last eight amino acids mediates a number of protein·protein interactions including binding to the χ subunit of the clamp loader (57–59). These χ·SSB interactions mediate the switch from DnaG primase to the core polymerase (60), stabilize the holoenzyme at the replication fork (46,47), and help coordinate leading and lagging strand synthesis (59).

To determine whether specific interactions between the clamp loaders and SSBs are important to preferential clamp loading on 3′-recessed ends, clamp closing was measured in reactions with non-cognate SSBs and was measured for a ‘χ-less’ γ complex. Clamp loading by RFC is unchanged on 3′DNA by the presence of SSB or RPA (Figures 3A and 6B, light blue) suggesting that specific protein·protein interactions between RFC and RPA are not required to load clamps on 3′DNA. However, the addition of SSB to 5′DNA did not inhibit PCNA closing to the extent that RPA did (Figures 3B and 6B, gray). Clamp loading on 5′DNA·RPA was 50-fold slower than 3′DNA·RPA, while clamp loading on 5′DNA·SSB was only 10-fold slower. This difference indicates that either SSB or RPA can slow clamp loading on 5′DNA, but RPA is required for robust inhibition of clamp loading, suggesting that RFC·RPA interactions may contribute to specificity. Surprisingly, two lines of evidence show that χ is not required for SSB-dependent inhibition of clamp closing on 5′DNA. Closing reactions on 5′DNA·SSB for both the complete clamp loader and the χ-less clamp loader occur at about the same rate as the slow, passive dissociation reaction (Figures 2B and 5A, gray) and RPA inhibits clamp loading on 5′DNA by γ complex (Figure 6A, gray). However, γ complex·SSB interactions do play a role in clamp loading on correct 3′-recessed ends. Closing reactions with 3′DNA·SSB (Figure 5A and B, light blue), were about 3-fold slower for the χ-less clamp loader when compared to wild-type γ complex and experiments substituting RPA for SSB on 3′DNA (Figure 6A, light blue) also yielded a 3-fold decrease in clamp closing rates for the wild-type clamp loader. These data indicate that species-specific γ complex·SSB interactions mediated by the χ subunit facilitate clamp loading on 3′DNA.

### Model for SSB-directed clamp loading on 3′-recessed ends

Taken together, our results show that SSBs strongly inhibit clamp loading on DNA of the wrong polarity (5′DNA) but not on the correct polarity DNA (3′DNA), and that specific protein·protein interactions between the clamp loaders and SSBs make a relatively small contribution to this specificity. Instead, the structure and dynamics of SSB·DNA complexes may make a larger contribution to specificity and this may be influenced by 5′phosphorylation. We propose a model by which SSB confers specificity for clamp loading at 3′-recessed ends in which the clamp loader·clamp complex initially binds the dsDNA near an ss/ds junction and slides to the junction displacing SSB from the junction. When the junction contains a 3′-recessed end, either the conformation of the SSB·DNA complex or the position and mobility of SSB on DNA is such that the clamp loader can more easily access the junction than when a 5′-recessed end is present. For the *E. coli* clamp loader, access to ss/ds junctions may depend at least in part on γ complex·SSB interactions mediated by the χ subunit. This would explain why clamp loading by the χ-less clamp loader is reduced by 3-fold on 3′-recessed ends and not strongly inhibited by SSB on unphosphorylated 5′-recessed ends. A 5′P at 5′-recessed ends may interact with SSB in some way to make it more difficult to move or alternatively affect the conformation of the SSB·DNA complex or position of SSB on DNA. Clamp loading by RFC is similar to the χ-less γ complex in that inhibition of clamp loading on 5′DNA is dependent on 5′-phosphorylation. This also suggest a mechanism in which the 5′P may affect the structure or dynamics of the 5′DNA·RPA complex to promote a conformation that blocks access of the clamp loader to the ss/ds junction or inhibits displacement of RPA from the junction to inhibit clamp loading.

Our model for the clamp loader displacing SSB agrees with the model proposed by Downey and McHenry to explain the effects of SSB on initiation complex formation (61), and extends this model to explain how SSB confers specificity for loading at 3′-recessed ends. Initial binding of clamp loaders to ds DNA is supported by fluorescence resonance energy transfer studies of the bacteriophage T4 clamp loader·clamp complex, which show that the clamp opens wide enough to accommodate the passage of ds DNA into the center of the ring (62,63). On the other hand, the bacteriophage T4 clamp was only opened by about 9 Å in the crystal structure of the ternary clamp loader·clamp·DNA complex, which is too small to accommodate ds DNA, therefore, the suggestion was made that the clamp loader could thread the clamp around ssDNA (15). For this to happen when SSB is bound to the ss template overhang, the clamp loader would have to slide SSB away from the ss/ds junction to free up enough ssDNA to pass through the clamp and this could be dependent on DNA polarity. Although this second scenario is possible, we favor the first for simplicity and because the clamp makes electrostatic interactions with the ds region of DNA that contribute to targeting it to DNA (30,64–66).

The 5′-phosphorylated DNA:DNA duplexes used in this study most closely model physiological structures that the clamp loaders would encounter in DNA gap repair where the 5′- and 3′-ss/ds DNA junctions of gaps must be distinguished. At a replication fork in *S. cerevisiae*, the primed template junction is composed of a RNA:DNA duplex at the 5′-end, and a DNA:DNA duplex at the 3′-end, while in *E. coli*, the p/t junction is an RNA:DNA duplex. These nucleic acid structures differ from the DNA:DNA structures used in this study in two main ways. First, an RNA:DNA duplex typically forms an A-DNA structure as opposed to a B-DNA structure for the DNA:DNA duplexes (67). Second, the 5′-end of the RNA primer contains a triphosphate instead of the monophosphates used in the DNA structures tested here. For the *E. coli* clamp loader, interactions with an A-form duplex may enhance clamp loading on 3′DNA as demonstrated for γ complex (30), but would not confer specificity for 3′- versus 5′-ends. For eukaryotic clamp loaders, if the primed template structure transitioned from A-form to B-form, this could confer additional specificity for loading clamps at 3′-recessed ends. Similarly, the presence of 5′-triphosphates could be more inhibitory toward clamp loading than 5′ monophosphates. Although either of these factors could enhance specificity for naked nucleic acid substrates, our results showed that SSBs gave the clamp loaders a strong preference for loading clamps at 3′-recessed ends. Given that the observed rates of both β and PCNA closing on 5′DNA·SSBs are within standard deviation of clamp closing/release rates in the complete absence of DNA (Figure 2B and (28,45)), it is unlikely that RNA primers or 5′-triphosphates could provide any greater inhibition of clamp loading on 5′-recessed ends.

The DNA structures used in these experiments were designed so that only a single SSB tetramer or RPA trimer could bind each ssDNA overhang based on the binding site sizes of SSB and RPA (68,69). *In vivo*, the SSBs oligomerize on ssDNA and additional molecules of SSB could have further effects on the structure and dynamics of SSB·DNA at the ss/ds junction. These effects could potentially magnify the difference in clamp loading on 3′ versus 5′DNA, but to do so clamp loading on 3′DNA·SSB would have to be enhanced because clamp loading on 5′DNA·SSB is already as slow as the passive clamp loader·clamp dissociation reaction.

## CONCLUSION

Clamp loaders perform the vital task of loading sliding clamps onto DNA for both replication and repair functions. These molecular machines must be able to bind the sliding clamp, find the correct position on DNA, and load the clamp for use by other proteins quickly and efficiently. A sliding clamp is required at ss/ds junctions with 3′-recessed ends in order to be an effective platform for DNA polymerases and other interacting enzymes. Therefore, clamp loaders must have a mechanism to target this location while simultaneously disregarding other DNA present in the cell. This work measures the DNA substrate specificity of γ complex and RFC and shows that SSBs prevent clamp loading on the wrong polarity of DNA by both clamp loaders. This SSB-mediated specificity is either partially, or entirely dependent on the presence of a 5′P group at the ss/ds junction. This study demonstrates a common general mechanism while uncovering subtle differences for DNA specificity of the *E. coli* and *S. cerevisiae* clamp loaders.

## SUPPLEMENTARY DATA

Supplementary Data are available at NAR Online.

SUPPLEMENTARY DATA

## ACCESSION NUMBER

PDB ID: 3U60.
