# Supplementary material for: The interplay of primer-template DNA phosphorylation status and single-stranded DNA binding proteins in directing clamp loaders to the appropriate polarity of DNA
Source: Nucleic Acids Res. 2014 Aug 26;42(16):10655–67. doi: 10.1093/nar/gku774 (PMC4176372; doi:10.1093/nar/gku774)
Supplement: SUPPLEMENTARY DATA [file supp_42_16_10655__index.html]

The interplay of primer-template DNA phosphorylation status and single-stranded DNA binding proteins in directing clamp loaders to the appropriate polarity of DNA — The interplay of primer-template DNA phosphorylation status and single-stranded DNA binding proteins in directing clamp loaders to the appropriate polarity of DNA — SUPPLEMENTARY DATA 

# The interplay of primer-template DNA phosphorylation status and single-stranded DNA binding proteins in directing clamp loaders to the appropriate polarity of DNA

## SUPPLEMENTARY DATA

**Files in this Data Supplement:**

- SUPPLEMENTARY DATA
