# Supplementary material for: The interplay of primer-template DNA phosphorylation status and single-stranded DNA binding proteins in directing clamp loaders to the appropriate polarity of DNA
Source: Nucleic Acids Res. 2014 Aug 26;42(16):10655–67. doi: 10.1093/nar/gku774 (PMC4176372; doi:10.1093/nar/gku774)
Supplement: SUPPLEMENTARY DATA [file supp_gku774_nar-01064-h-2014-File008.pdf]

SUPPLEMENTARY DATA

TABLES

Table S1. Comparison of rates of PCNA-MDCC release from RFC (Release rates) with rates of the slow phase of the closing assay (Closing rates)

	**– RPA**		**+ RPA**	
**DNA structure**	Release rates	Closing rates	Release rates	Closing rates
	k_{obs} (s^{-1})	k_{obs} (s^{-1})[a]	k_{obs} (s^{-1})	k_{obs} (s^{-1})[a]
**3'DNA**	0.52 ± 0.3	0.44 ± 0.01	0.25 ± 0.02	0.33 ± 0.07
**5'DNA**	0.27 ± 0.01	0.27 ± 0.03	0.09 ± 0.01	0.09 ± 0.01[b]

[a]Values for k_{obs2} from Table 2 in the main manuscript.

[b]The decrease in reactions with 5'DNA•SSB is monophasic and likely passive dissociation rather than biphasic for active clamp loading/closing followed by RFC-PCNA dissociation.

Table S2. Comparison of clamp loading on 5'-phosphorylated DNA substrates with and without SSBs.

	β loading closing (s^{-1})				PCNA closing rates (s^{-1})		
	No SSB	SSB + χ	SSB – χ	RPA	No RPA	RPA	SSB
3'DNA	4.6 (2.4)*	5.7 (95)	1.8 (30)	1.6 (13)	3.3 (2.5)	4.4 (49)	4.6 (10)
5'DNA	1.9 (1)	0.06 (1)	0.06 (1)	0.12 (1)	1.3 (1)	0.09 (1)	0.45 (1)

* Rates relative to the rate of 5'DNA are given in parentheses to show the fold change.

FIGURES

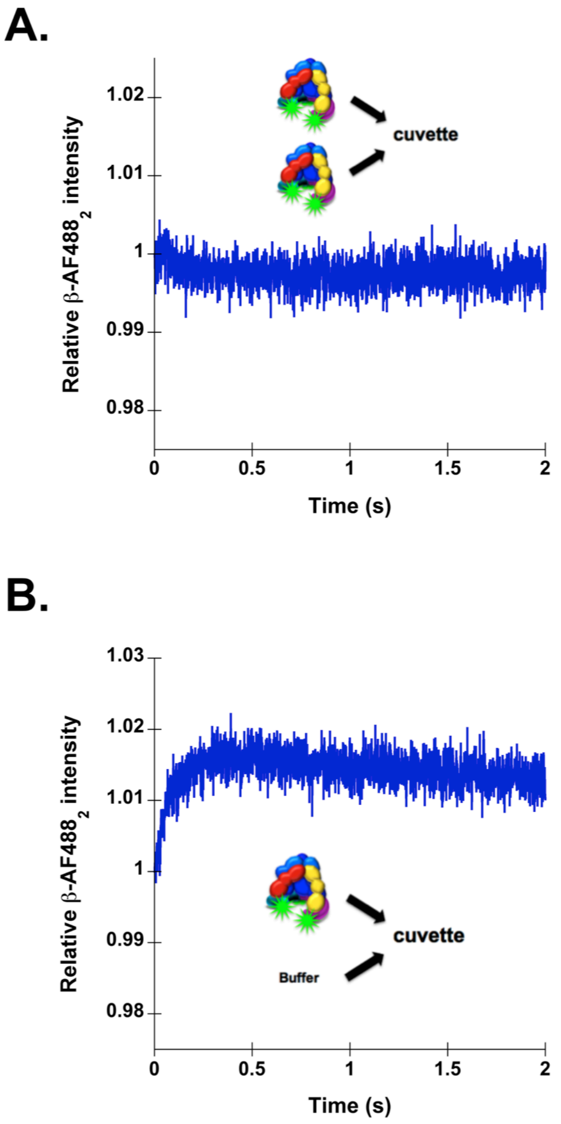

Figure S1. Dilution of the clamp loader•clamp complex results in a small fluorescence increase. A, Two solutions containing 20 nM γ complex, 20 nM β, and 0.5 mM ATP were mixed together in the stopped-flow, and fluorescence was measured as a function of time. B, A solution containing 40 nM γ complex, 40 nM β, and 0.5 mM ATP was mixed with buffer, and fluorescence was measured as a function of time.

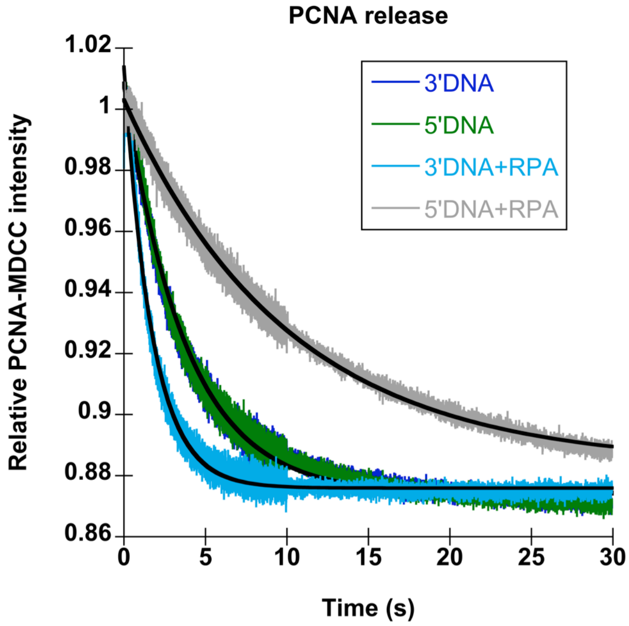

Figure S2. Clamp release by RFC. The observed rates of clamp release for PCNA were measured using a PCNA-MDCC mutant (Marzahn, submitted for publication). Reactions were performed as described in Fig 1B with final concentrations of proteins as described in Fig 3A. The black lines through the traces represent empirical fits using Eq. 1, with observed rates reported in Table S1. PCNA release experiments were performed for 3'DNA (dark blue), 5'DNA (green), 3'DNA•RPA (light blue), and 5'DNA•RPA (grey).
